# Latent Rheumatic Conditions

**Published:** 1906-04

**Authors:** J. R. Phelan

**Affiliations:** Oklahoma City, Editor Oklahoma Medical News


### Latent Rheumatic Conditions

By J. R. PHELAN, M.D., Oklahoma City,
Editor Oklahoma Medical News.

[*Oklahoma Medical News.*]

It is during the spring months more particularly that the physician is called upon to treat patients, who though not ill enough to be in bed, are not at all well. Their appetite is capricious, they sleep indifferently, or even if they sleep soundly they are not refreshed, and in the morning they are almost as fatigued and ill at ease as was the case on retiring. Upon awakening there is frequently an aching sensation in the loins, sometimes in the lower limbs, which may partially wear off as the day progresses, but there is at all times a vague, undefined, uneasy painful feeling.

The symptoms are very much like those experienced in malaria, but the causes often are entirely different hence a different treatment is necessary. This condition arises from the fact that in the spring the eliminative functions do not present their usual activity, owing to the torpor and locked-up secretions which have existed during the winter months, when the skin neglects its duties and the kidneys are overworked. If the condition remains neglected the probable result will be sooner or later a pronounced attack of rheumatism or grippe in one or another of its forms. All that is needed to induce such an attack is a sudden change in the weather or the exposure on the part of the patient to cold or wet or to a combination of both. This is due to a latent rheumatic diathesis to which every adult is liable.

The necessity of a powerful eliminant in every prescription for rheumatism and grippe is self-evident. While antipyretics and antiperiodics may slightly stimulate the excretions and relieve congestion thereby controlling certain features of the disease, a complete cure cannot be expected until the poisons are thoroughly eliminated from the system and the diseased organs enabled to resume normal functions. In the treatment of all rheumatic, neuralgic and grippy conditions, tongaline, by promoting the absorptive powers of the various glands which have been clogged, and by its stimulating action upon the liver, the bowels, the kidneys and the skin, will relieve the pain, allay the fever, eliminate the poisons, stimulate recuperation and prevent sequelæ.

